# Fabrication and Dose–Response Simulation of Soft Dual-Sided Deep Brain Stimulation Electrode

**DOI:** 10.3390/mi16080945

**Published:** 2025-08-18

**Authors:** Jian Zhang, Bei Tong, Changmao Ni, Dengfei Yang, Guoting Fu, Li Huang

**Affiliations:** Wuhan Neuracom Technology Development Co., Ltd., Wuhan 430074, China; zhangjian@neuracom.com.cn (J.Z.); tongbei@neuracom.com.cn (B.T.); nichangmao@neuracom.com.cn (C.N.); yangdengfei@neuracom.com.cn (D.Y.); fuguoting@neuracom.com.cn (G.F.)

**Keywords:** MEMS, DBS electrode, FEM, dose–response analysis

## Abstract

A 16-channel dual-sided flexible electrode based on a polyimide substrate was designed and fabricated using micro-electromechanical system (MEMS) technology. The electrode exhibited an average impedance of 5.9 kΩ at 1 kHz and a charge storage capacity (CSC) of 10.63 mC/cm^2^. Concurrently, a three-dimensional finite element model incorporating electrical stimulation and micromotion-induced damage was established. The simulation results demonstrated that the implantation trauma caused by the bilateral electrode was significantly lower compared with silicon-based and cylindrical electrodes, while also enabling directional stimulation. Furthermore, leveraging the design of experiments (DOE) methodology, a multivariate regression model was developed to investigate the influence of key stimulation parameters—namely, current amplitude, frequency, and pulse width—on the volume of tissue activated (VTA). The results indicated that the regression model provided accurate predictions of VTA (R^2^ = 0.912). Among the parameters, current amplitude and pulse width exerted a statistically significant influence on VTA size (*p* < 0.001), whereas the effect of frequency was comparatively minor (*p* = 0.387 > 0.05). This study presents the first successful fabrication and comprehensive dose–response analysis of a flexible bilateral DBS electrode. Its attributes of low implantation trauma, multi-channel capability, and directional stimulation offer a novel paradigm for precise neuromodulation. Additionally, the established stimulation parameter–VTA response model provides a robust theoretical foundation for optimizing therapeutic parameters in subsequent clinical applications.

## 1. Introduction

Deep brain stimulation (DBS) is an FDA-approved neuromodulation therapy that has been widely employed to treat neurological disorders such as Parkinson’s disease, essential tremor, and epilepsy [1,2,3,4].

Although studies have confirmed the efficacy of DBS in relevant indications, the precise biological mechanisms remain unclear. The therapeutic process involves complex physiological interactions among implanted electrodes, stimulation parameters, and neural tissue [5]. To maximize clinical outcomes while minimizing adverse effects, precise control over the electric field distribution is critical [6]. Conventional cylindrical electrodes generate spatially spherical electric fields that often extend beyond the target region. Human brain mapping reveals that target neuroanatomy does not conform to regular shapes like spheres and is mismatched with the ellipsoidal or spindle-shaped fields produced by parameter adjustments. Therefore, this study proposes two approaches to mitigate side-effect risks: directional stimulation and miniaturization. Directional stimulation modulates the field distribution through geometrically engineered electrode contacts or parameterized programming of stimulation channels. Miniaturization aims to position electrodes entirely within target nuclei while enabling precise on/off control of stimulation channels via integration with imaging-guided programming.

Recent advances in MEMS technology have provided reliable pathways for functional integration and miniaturized low-trauma neural electrodes [7], exemplified by the Utah array and Michigan probe [8,9]. However, silicon-based microelectrodes targeting deep brain regions typically exceed 5 mm in length, rendering them prone to brittle fracture during implantation [10,11,12] while simultaneously causing tissue damage. Electrode brittleness and chronic issues—such as fretting damage and inflammatory responses resulting from mechanical mismatch between materials and brain tissue—constitute major bottlenecks in the clinical translation of traditional neural microelectrodes [13,14,15]. In contrast, flexible electrodes demonstrate the potential to overcome the mechanical limitations of rigid devices. Compliant materials like PDMS, hydrogels, and polyimide achieve elastic modulus matching, significantly reducing glial scar formation [16,17]. Among these, polyimide has emerged as an ideal implant material due to its flexibility, chemical inertness, corrosion resistance, and MEMS process compatibility [18], leading to its widespread adoption in electrode applications [19,20,21].

Current parameter optimization in DBS therapy primarily relies on empirical clinical observations of patient responses with various stimulation parameter combinations. Postoperative follow-up data indicate an average of four programming sessions per patient within the first year. The finite element method (FEM) provides data-driven support for optimizing DBS programming while reducing trial-and-error costs, offering advantages in visualizing the effects of multiple stimulation parameters and electrode configurations. A widely adopted approach for evaluating stimulation efficacy is the volume of tissue activated (VTA) model [22,23,24,25], which delineates activation boundaries using different neural excitability metrics, primarily including axon cable modeling [26], activation function thresholds [27], and electric field norm methods [28].

Addressing clinical demands for multi-channel capability, low implantation trauma, directional stimulation, and programming optimization in DBS therapy, this study designed and fabricated a flexible polyimide double-sided electrode using MEMS technology. The 16-channel device enables both low-trauma implantation and bidirectional directional stimulation. By establishing an electrical stimulation model, we visualized spatial electric field distributions in brain tissue and quantified the volume of tissue activated (VTA) responses with diverse stimulation parameter combinations. Key determinants of VTA were systematically analyzed to establish quantitative mappings between stimulation parameters and therapeutic outcomes, thereby informing precise programming and clinical parameter optimization.

## 2. Materials and Methods

### 2.1. Electrode Structure Design and Manufacturing

**Figure 1:**
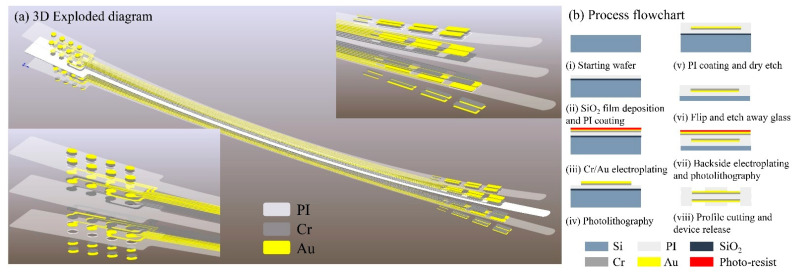
(**a**) Three-dimensional exploded diagram of electrode; (**b**) process flowchart of electrode.

The DBS electrode features 16 channels arranged in a 2 × 4 array on both the front and back surfaces. Figure 1 illustrates the fabrication process flow and provides a multilayer schematic of the electrode structure.

The fabrication begins by depositing 1 μm of SiO_2_ via PECVD on an 8-inch wafer, followed by spin coating a 5 μm polyimide (PI) flexible substrate. A 50 nm Cr adhesion layer is electroplated before depositing a 200 nm Au front-side metallization layer. After spin coating a second PI insulating layer, partial etching exposes contact electrodes. Upon completing front-side metallization and insulation, the front side is bonded to a secondary glass carrier. The primary carrier is then dissolved by HF wet-etching to expose the PI backside. Following flip-chip bonding, identical processing steps pattern the backside metallization and perform window opening. Finally, the PI flexible electrode is released from the substrate.

### 2.2. Electrode Performance Characterization

To evaluate the electrical performance of the dual-sided DBS electrode, electrochemical testing was performed in phosphate-buffered saline (PBS). Cyclic voltammetry (CV) determined the electrode’s safe water window, while electrochemical impedance spectroscopy (EIS) quantified electrode–electrolyte interface characteristics. These measurements verified the charge injection capacity (CIC) and polarization potential safety, thereby ensuring reliability for future in vivo experiments.

A three-electrode system was employed for cyclic voltammetry (CV) and electrochemical impedance spectroscopy (EIS) measurements. The system utilized a Ag/AgCl reference electrode and a platinum (Pt) counter electrode (model LEDONLAB R0303; Pt purity > 99.99%). The electrolyte consisted of 10× PBS cell buffer (1.37 M NaCl, 27 mM KCl, 100 mM Na_2_HPO_4_, and 18 mM KH_2_PO_4_; pH 7.4) to mimic the physiological tissue environment. During testing, the reference electrode, the counter electrode, and the flexible device under test (DUT) electrode were immersed in the electrolyte solution, maintaining an inter-electrode distance of 2 cm. An electrochemical workstation (DH7000C, Donghua Testing Technology Co., Ltd., Jingjiang, China) was used for all measurements.

- CV parameters: A triangular waveform with a scan rate of 50 mV/s was applied to establish the electrode’s safe water window.
- EIS parameters: Impedance spectra were acquired over a frequency range of 1 Hz to 100 kHz with an applied sinusoidal AC signal amplitude of 10 mV RMS to characterize the electrode’s impedance changes across frequencies.

### 2.3. Electrical Stimulation Model and Dose–Response Analysis

This section establishes a computational reference model for the dual-sided DBS electrode–brain tissue interface and investigates the stimulation–response relationship between electrical parameters and neural activation. First, we developed a finite element model (FEM) based on quasi-static Maxwell’s equations and the steady-state Pennes bioheat equation to simulate the electric field and temperature distributions in brain tissue. Second, the volume of tissue activated (VTA) was calculated using the activating function threshold method. Finally, a design of experiments (DOE) approach systematically quantified the effects of key stimulation parameters—current amplitude, frequency, and pulse width—on VTA and morphology. This analysis established quantitative criteria for stimulation–response relationships, providing a theoretical basis for precise neuromodulation during preoperative planning and intraoperative parameter optimization.

**Figure 2:**
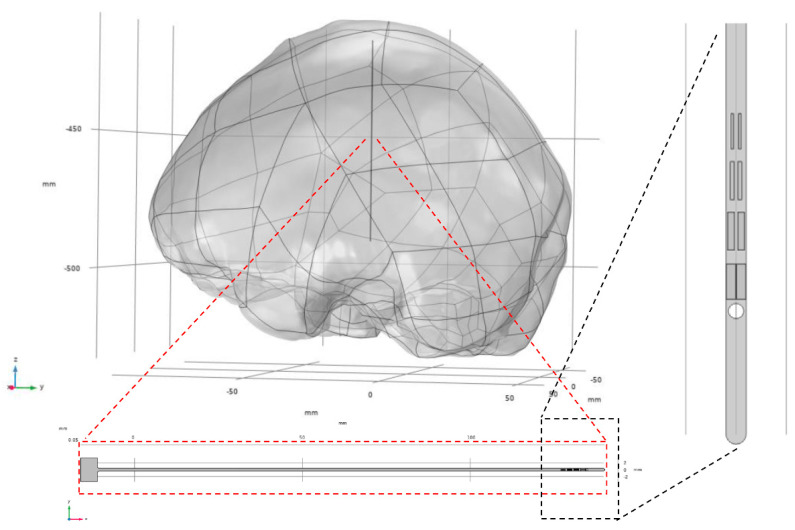
Assembly relationship between electrodes and brain tissue model.

A geometric model of the cranial structure was reverse-engineered based on desensitized cranial CT data using MIMICS (Materialise NV, Leuven, Belgium, 2018) and Geomagic Wrap (3D Systems, Inc., Rock Hill, SC, USA). The resulting brain tissue model was then imported into the COMSOL Multiphysics 6.2 (COMSOL AB, Stockholm, Sweden) preprocessing environment. Subsequently, a three-dimensional electrode model was constructed within COMSOL based on the electrode layout design. Figure 2 illustrates the assembled model, depicting both the electrode and brain tissue components, along with their spatial configuration.

**Figure 3:**
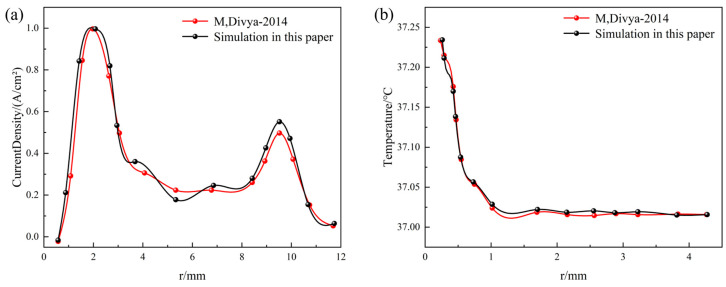
Numerical method verification, comparing with M, Divya [28] (**a**) axial current density distribution comparison and (**b**) axial temperature distribution comparison.

First, to validate the numerical simulation methodology, we reproduced the simulation work from reference [28]. Using the geometric parameters and electrical/thermal properties of tissue reported therein, a computational model was established and executed, the tissue physical parameters is shown in Table 1. Electric field strength data along the electrode’s axial cross-section and temperature distribution were extracted and are plotted in Figure 3. The results demonstrate close agreement between our simulated electric field distribution and the reference data. The peak temperature rise of 0.2425 °C shows a 2.1% deviation from the literature value. This confirms that our numerical approach accurately characterizes the electrothermal coupling behavior between the electrode and tissue.

**Figure 4:**
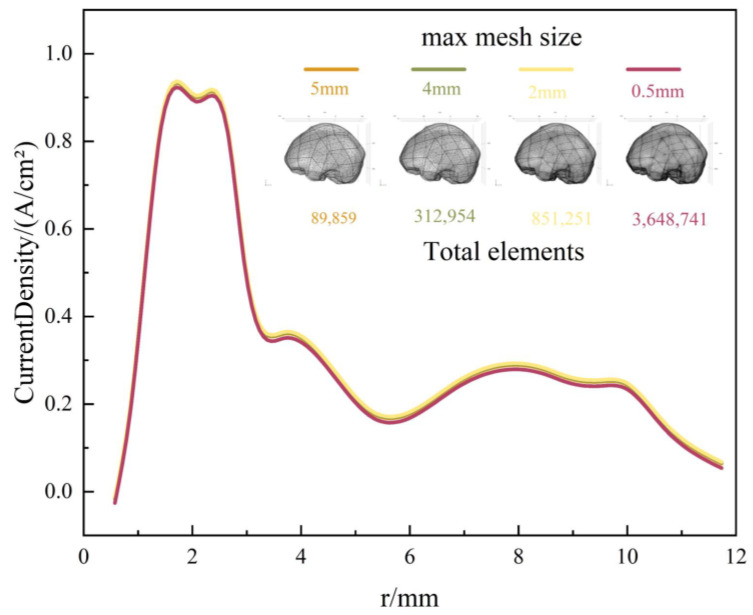
Current density of FE model with four maximum mesh sizes.

Second, following the validation of the numerical methodology, mesh independence verification was conducted for the electrode–brain tissue electrostimulation baseline model. Maximum mesh sizes of 5 mm, 4 mm, 2 mm, and 0.5 mm were selected. Figure 4 presents the surface current density profiles along the electrode length for models employing these four different mesh sizes. As the mesh undergoes progressive refinement, the current density curves exhibit a convergence trend, overlapping increasingly. However, the significant increase in the total element count associated with finer meshes leads to a substantial rise in the computational time. In summary, employing a maximum mesh size of 2 mm ensures the convergence of the numerical results while keeping the computational time within reasonable limits. Therefore, this mesh size (2 mm) was adopted for all subsequent multi-case simulations.

Following the validation of the numerical methods and mesh independence verification, the baseline model for dual-sided DBS electrode–brain tissue electrostimulation was established. Using the design of experiments (DOE) methodology, simulation scenarios were systematically configured to investigate the multi-physics responses of brain tissue under diverse stimulation parameters. This approach aimed to develop a semi-analytical, semi-empirical dose–response assessment framework for guiding stimulation parameter optimization and precise spatial control of activation volumes.

Drawing upon established DBS clinical stimulation parameters [29,30], the key stimulation parameters—specifically, current amplitude (I), pulse width (P_w_), and frequency (f)—were selected as the experimental factors. The number of levels for these three factors was set at 4, 3, and 4, respectively. Employing a full factorial design, a total of 48 simulation cases were configured, with the volume of tissue activated (VTA) serving as the response variable. The specific experimental parameter settings are listed in Table 2.

## 3. Results and Discussion

### 3.1. Electrochemical Performance of Electrodes

**Figure 5:**
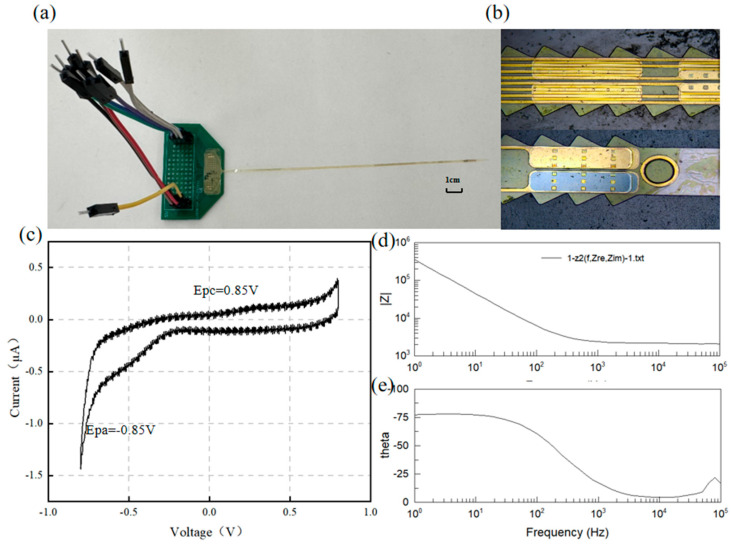
(**a**) Electrode and PCB, (**b**) surface morphology, (**c**) electrode CV curve, and (**d**,**e**) electrode EIS test results.

The electrochemical impedance spectroscopy (EIS) and cyclic voltammetry (CV) characteristics of the electrode are critical determinants of the functional electrophysiological signal recording quality. The CV curves and impedance of the flexible electrode were measured using a three-electrode electrochemical workstation. As shown in Figure 5e, a pair of characteristic peaks was evident at 0.85 V and −0.85 V during the electrode activation phase. The charge storage capacity (CSC) of the electrode, calculated by integrating the voltage over one complete cycle, was determined to be 10.63 mC/cm^2^.

**Figure 6:**
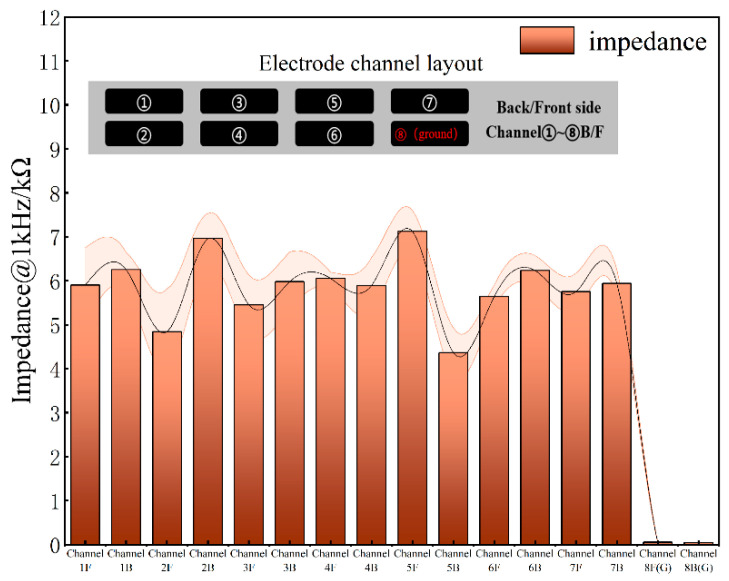
Electrode impedance of all channels at 1kHz.

For implantable neural electrodes, maintaining low interfacial impedance is essential to mitigate electrode polarization and reduce energy consumption in stimulation circuitry [31]. Given typical pulse durations of 1 ms, an electrode impedance of 1 kHz serves as a critical evaluation metric [32]. Figure 5 displays the frequency-dependent impedance magnitude and phase angle characteristics of the DBS electrode, showing an average impedance of ~5.9 kΩ at 1 kHz. Figure 6 shows the impedance of all the channels. The phase angle trend in Figure 5e indicates dominant capacitive behavior, with the impedance magnitude being inversely proportional to frequency.

Previous studies indicate that a charge density of 10 µC/cm^2^ is sufficient to evoke electrically evoked neural responses without damaging neural tissue [33]. Although DBS electrodes may exhibit an adequate charge injection capacity under in vitro conditions, their actual charge delivery capability often diminishes significantly following implantation into brain or spinal tissue, deviating substantially from linear predictions [34]. Electrochemical testing demonstrated that the DBS electrodes developed in this study are suitable for neural stimulation applications.

### 3.2. Electrode Implantation Injury and Directionality Comparison

**Figure 7:**
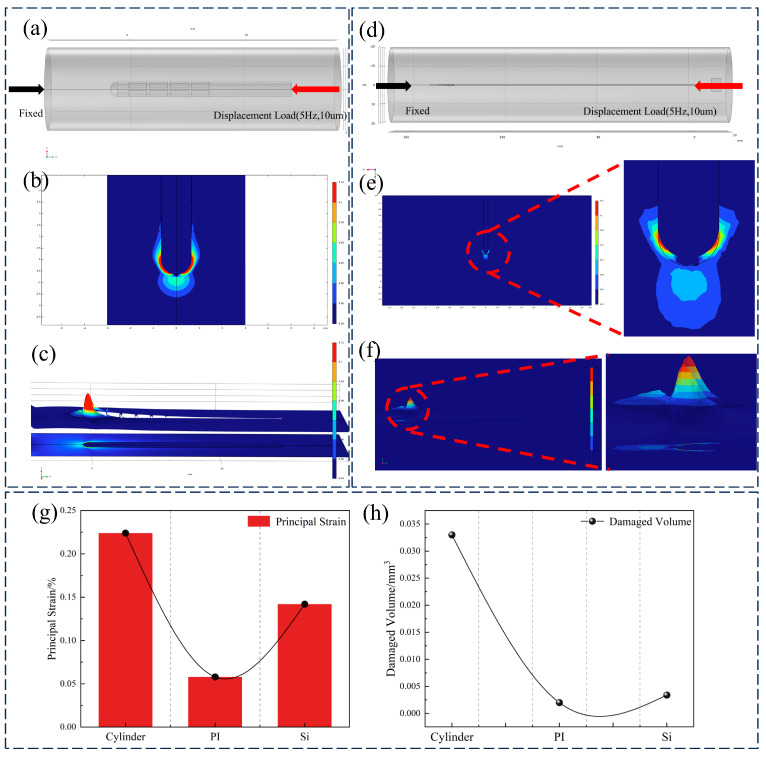
(**a**) Cylindrical electrode micromotion injury model, (**b**) sagittal plane strain cloud map, (**c**) strain contour cloud map, (**d**) double-sided electrode micromotion injury model, (**e**) sagittal plane strain cloud map, (**f**) strain contour cloud map, and (**g**,**h**) comparison of maximum principal strain and injury volume of cylindrical electrode, double-sided flexible electrode, and silicon-based electrode.

Micromotion-induced damage has been identified as a primary contributor to tissue encapsulation and electrode failure [35]. Among various sources of brain micromotion, longitudinal displacement resulting from physiological activities such as respiration and cardiac pulsation exerts the most significant effect [36]. Consequently, this study focused on micromotion injury induced by longitudinal displacement. To compare post-implantation micromotion-induced damage, finite element simulations were conducted for the proposed flexible double-sided electrode, a conventional cylindrical electrode, and a typical silicon-based microelectrode. Based on experimental parameters reported in previous studies [37], a sinusoidal displacement with an amplitude of 10 μm and a frequency of 4 Hz was applied to the top surface of the electrodes to simulate micromotion over a total duration of 0.5 s. The maximum principal strain in brain tissue was employed as the damage metric [38], with a 5% strain threshold [39,40] used to define damage isosurfaces during post-processing. The volume of tissue exceeding this strain threshold was quantified for each electrode type. The cylindrical electrode exhibited the highest maximum principal strain (21.454%), compared with 14.52% for the silicon-based electrode and 5.31% for the double-sided electrode. This trend was consistent with the volume of tissue damage, wherein the flexible double-sided electrode demonstrated a 46.25% reduction in damage volume relative to the cylindrical electrode, as illustrated in Figure 7c,f.

Compared with traditional cylindrical electrodes and silicon-based microneedles, the polyimide-based double-sided electrode significantly reduces mechanical mismatch and tissue damage. While cylindrical electrodes feature simple structures, their relatively large cross-sectional area exacerbates tissue trauma during implantation, typically leading to gliosis and signal degradation. Silicon microneedles enable high-density integration, yet their brittleness and high stiffness relative to brain tissue frequently induce substantial damage. In contrast, the flexible double-sided electrode proposed in this study exhibits enhanced mechanical compliance with surrounding tissue, thereby mitigating chronic inflammation and micromotion-induced injury. The simulation results (Figure 7) indicate that under micromotion loading, both flexible and cylindrical electrodes develop stress concentrations near the electrode tip; however, the maximum tissue strain induced by the flexible electrode exhibits a 77% reduction relative to cylindrical electrodes and a 64.3% reduction relative to silicon electrodes. Furthermore, the deliberately flattened profile of the proposed electrode yields a smaller effective contact area than an equivalent-width cylindrical electrode, thereby reducing acute implantation trauma.

The activation threshold-based method is widely employed to estimate the volume of tissue activated (VTA) [41]. The activation threshold is defined as the minimum electric field intensity required to elicit an action potential and is typically determined through simulations using cable models of specific neuronal types. In this study, a tissue activation threshold of 20 V/m was applied to evaluate neural activation [42,43,44,45].

**Figure 8:**
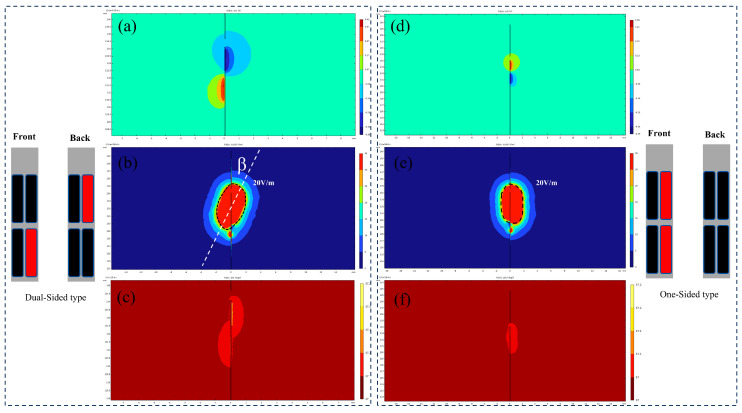
(**a**–**c**) Double-sided electrode potential, field intensity, and temperature isosurfaces. (**d**–**f**) Single-sided electrode potential, field intensity, and temperature isosurfaces.

Figure 8 compares the electric field profiles and temperature distribution contours between dual-sided and single-sided electrodes under identical stimulation parameters (0.8 mA, 100 Hz, and 100 μs rectangular pulses). The sagittal planes of the computational models were selected for visual comparison. Dual-sided stimulation demonstrated distinct directional field modulation: under a bipolar configuration, the tissue activation threshold isosurface exhibited an elliptical geometry with a 28.6° inclination angle (β) relative to the electrode axis. Using 20 V/m as the activation threshold isosurface, the calculated VTA values were as follows:

- Single-sided electrode: 16.31 mm^3^;
- Dual-sided electrode: 24.67 mm^3^.

**Figure 9:**
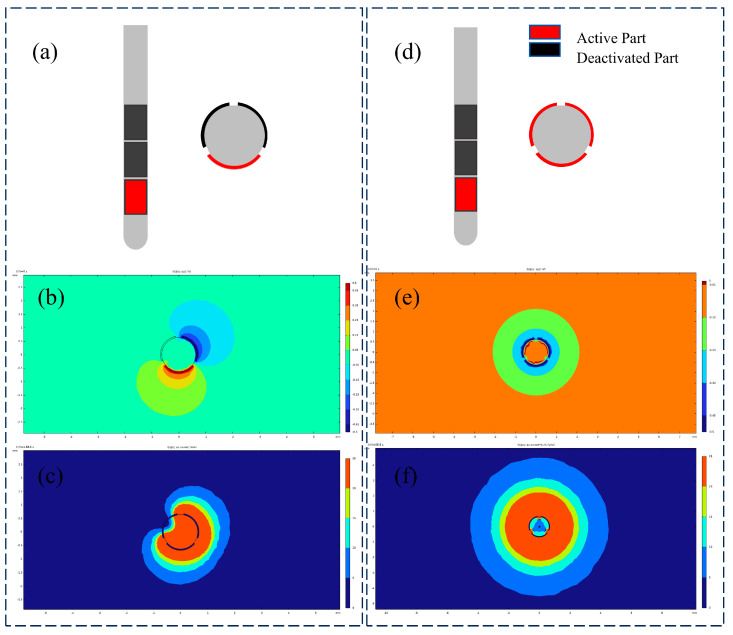
(**a**) Directional stimulation with cylindrical electrodes, (**b**) directional stimulation potential contour surface, (**c**) directional stimulation field intensity contour surface, (**d**) omnidirectional stimulation with cylindrical electrodes, (**e**) omnidirectional potential contour surface, and (**f**) omnidirectional field intensity contour surface.

Additional simulations of the electric field distributions of cylindrical electrodes in tissue are presented in Figure 9, comparing directional and omnidirectional stimulation modes under identical stimulation parameters (0.8 mA, 100 Hz, and 100 μs rectangular pulses). In directional stimulation mode, the cylindrical electrode generated a constrained, dumbbell-shaped potential field with distinctly oriented electric field isosurfaces. Regions of minimal electric field (blind zones) existed around inactive contacts, resulting in a tissue activation volume of 29.97 mm^3^. Conversely, omnidirectional stimulation produced a spherically symmetric potential distribution with uniformly radial electric field isosurfaces, exhibiting no directional preference. The resulting activation volume of 44.25 mm^3^ was significantly larger than that achieved in directional mode, though at the expense of spatial precision.

To evaluate the spatial performance of the proposed double-sided electrode, the electric field spatial coverage of both electrode types was compared under two stimulation modes. The results demonstrated that for cylindrical electrodes, directional stimulation achieved 63.4% of the spatial coverage obtained with omnidirectional stimulation. In contrast, the double-sided electrode attained 75.62% spatial coverage in directional mode relative to single-sided stimulation. Both electrode configurations exhibited sufficient blind zones to prevent unintended activation of irrelevant nuclei. Furthermore, the double-sided design enabled multi-channel recording and stimulation while efficiently utilizing the limited substrate area without increasing probe width. Compared with single-sided electrodes, this configuration facilitated directional stimulation with efficacy comparable to mainstream commercial electrodes.

### 3.3. Dose–Response Analysis Prediction Model

Analysis of variance (ANOVA) was conducted on 48 simulation conditions to verify their statistical significance. Based on the simulation data (shown in Appendix A), a multiple regression model was established to analyze the parameter main effects and interaction effects. Both current amplitude and pulse width exerted significant main effects on the volume of tissue activated (VTA) (*p* < 0.05), with the VTA increasing monotonically with amplitude and pulse width elevation, consistent with established neurophysiological principles of spatial electrical stimulation. Additionally, their interaction was statistically significant (*p* < 0.05), indicating synergistic regulation of VTA. In contrast, stimulation frequency exerted no significant effect on the VTA (*p* = 0.387) within the tested range (50–130 Hz), and its interactions with other parameters were non-significant. From an electric field distribution perspective, frequency primarily modulates temporal rather than spatial characteristics of stimulation.

**Figure 10:**
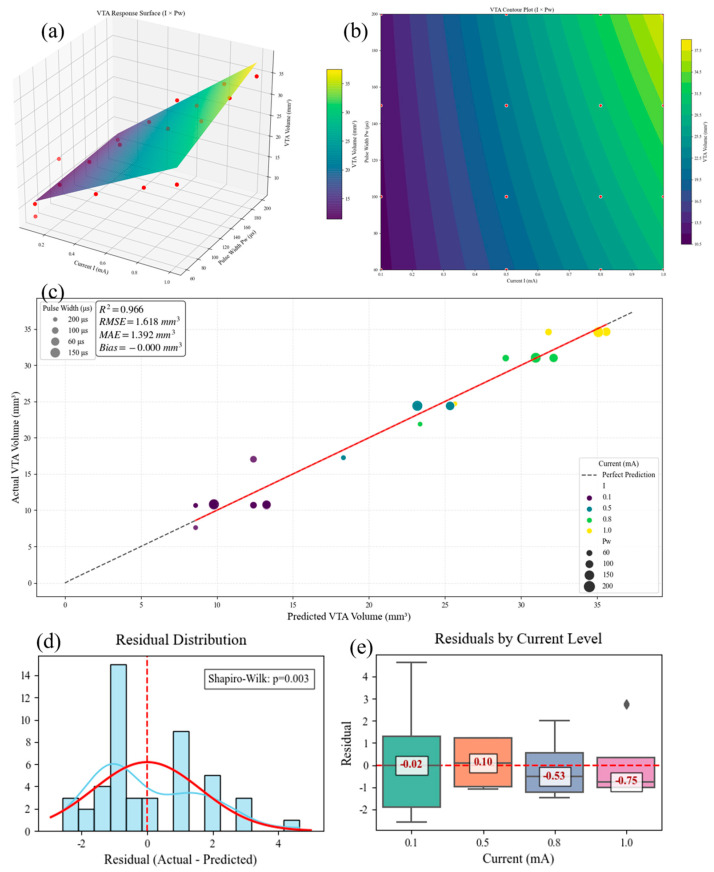
(**a**) Model response surface, (**b**) contour plot of different parameters, (**c**) model prediction effect, (**d**) residual normality diagnostic plot, and (**e**) current parameter residual band plot.

Further regression analysis yielded a predictive equation for the VTA. Employing ordinary least-squares (OLS) fitting, we present the resulting response surface, contour plot, and residual diagnostic plots in Figure 10. The model exhibited a root-mean-square error (RMSE) of 1.27 mm^3^ and a coefficient of determination (R^3^) of 0.912, indicating an explanation of 91.2% of the variance and a mean prediction error of 1.27 mm^3^. Residual normality was confirmed using the Shapiro–Wilk test (*p* > 0.05). The final functional relationship between stimulation current (I), pulse width (PW), and VTA is expressed as

(1) $$VTA=9.31+15.21I+0.0014P_{w}+0.0653\cdot \left(I\cdot P_{w}\right)$$

where VTA is the volume of tissue activated (mm^3^), I is the stimulation current (mA), and P_w_ is the stimulation pulse width (μs).

The VTA prediction model derived in another study [43] approximated the activated tissue volume as spatial spheres. To facilitate the comparison of response magnitudes under varying stimulation parameters, that study introduced the concept of an effective VTA radius. The applicability of this model is confined to monopolar and bipolar stimulation using rectangular pulses. By substituting Equation (2) into the spherical volume formula, Equation (3) is obtained, as follows:

(2) $$r={\left(\frac{P_{w}}{90\;\mu s}\right)}^{0.3}\cdot \sqrt{0.72\cdot \frac{I}{165}}$$

(3) $$VTA={\left(\frac{P_{w}}{90\;\mu s}\right)}^{0.6}\cdot 0.96\cdot \pi \cdot \frac{I}{165}$$

where r is the radius of VTA (mm), I is the stimulation current (mA), and P_w_ is the stimulation pulse width (μs).

Comparing Equations (1) and (3), the prediction model from the literature [43] exhibits a nonlinear formulation. Within this model, the influence of pulse width (P_w_) on VTA demonstrates an exponential decay of marginal effects. Furthermore, the literature model does not account for parameter interaction effects.

In contrast, the prediction model proposed in this study (Equation (1)) features a linear formulation that explicitly incorporates parameter interaction terms, resulting in a more concise mathematical expression.

**Figure 11:**
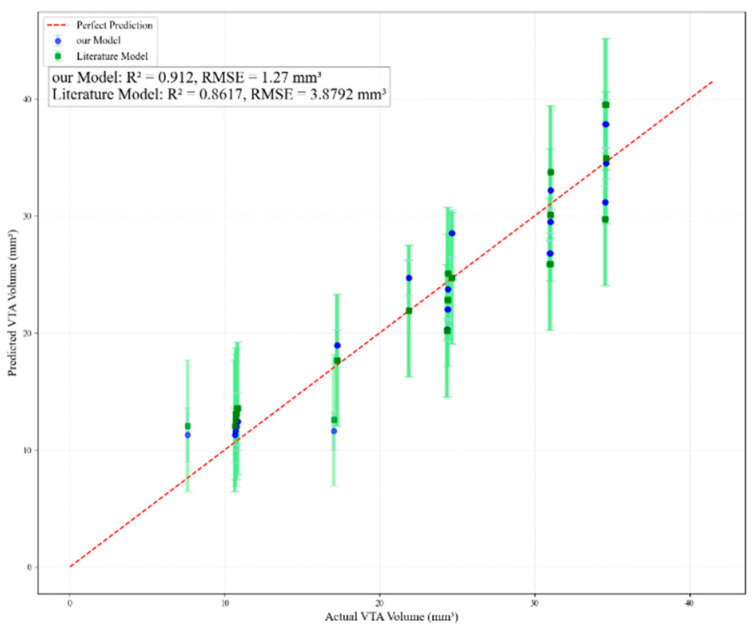
Comparison of prediction effects between this paper’s model and other scholars’ models.

To evaluate the predictive performance of both models, the simulation results generated in this study served as the validation dataset. The predictive outcomes of both models are plotted in Figure 11. The literature model [43] yielded a root-mean-square error (RMSE) of 3.8792 mm^3^ and a coefficient of determination (R^2^) of 0.8617. The results clearly indicate that the predictive performance of the model developed in this study is superior to that of the model presented in reference [43].

Dose–response analysis revealed that stimulation current directly determines stimulation intensity and exhibits a positive correlation with the volume of tissue activation (VTA). The influence of pulse width on VTA increases with higher stimulation current intensities, while its independent regulatory effect is less pronounced compared with stimulation current. Based on these quantitative relationships, personalized therapeutic paradigms can be designed to achieve mode switching (e.g., selecting high-current protocols versus high-efficiency protocols) while minimizing the total energy delivered (TEED).

In clinical practice, physicians can leverage patient-specific MRI-derived brain atlases to mathematically derive stimulation parameters based on the target region size. This approach offers actionable solutions for optimizing postoperative stimulation parameter programming, thereby minimizing stimulation-induced side effects. Traditional parameter adjustment in deep brain stimulation (DBS) therapy relies heavily on clinicians’ empirical experience and necessitates prolonged trial-and-error processes. In contrast, the presented predictive model, grounded in extensive simulation data, significantly reduces parameter optimization time while simultaneously lowering trial-and-error costs.

The Medtronic electrode employs a cylindrical lead with segmented contacts (4–6 directional rings) requiring a 1.27 mm diameter insertion [46]. Boston Scientific Vercise uses an eight-contact cartridge design (diameter = 1.3 mm) for current steering [47]. In contrast, our MEMS-fabricated polyimide electrode achieves 16 channels on a 0.2 mm thick substrate (width = 0.8 mm), reducing the cross-sectional area by 62% while doubling the channel density (Figure 1a).

The dual-sided designed DBS electrode developed in this study exhibits limitations in long-term implantation stability, while the established electrical stimulation baseline model requires further refinement in generalizability and precision. Specifically, several key modeling assumptions warrant explicit acknowledgment:

(1) Steady-state thermal approximation in the Pennes bioheat equation neglects transient effects during pulsed stimulation;

(2) Isotropic tissue properties with uniform conductivity (0.33 S/m) and thermal conductivity (0.5 W/m·K) ignore the anisotropic nature of neural pathways;

(3) Static activation threshold: The 20 V/m criterion disregards spatial variations in neuronal density.

Consequently, electrode displacement in our model only translates the VTA without altering its volume or morphology, failing to reflect real-world targeting errors.

For finite element model improvements, future work will incorporate dynamic thermal modeling and tissue nonlinearity by calibrating frequency-dependent conductivity/permittivity and diffusion tensor imaging (DTI)-derived anisotropic properties through in vitro experiments to enhance computational accuracy.

Regarding electrode stability, long-term implantation experiments in PD rat models will be conducted, involving periodic monitoring of electrode integrity and behavioral responses.

## 4. Conclusions

This study designed and fabricated a polyimide-based 16-channel dual-sided flexible DBS electrode using MEMS technology. First, 3D finite element models simulating electrical stimulation and micro-motion damage demonstrated that the dual-sided electrode significantly reduces implantation-induced tissue damage compared with silicon-based and cylindrical electrodes while enabling directional stimulation. Second, a multivariate regression model based on the design of experiments (DOE) method was established to investigate the effects of stimulation parameters (current amplitude, frequency, and pulse width) on the volume of tissue activation (VTA). The results indicate that the regression model accurately predicts VTA.

Future work will focus on validating the long-term implantation stability of DBS electrodes and exploring the feasibility of closed-loop control integration. Specifically, the bidirectional recording and stimulation capability of the dual-sided electrode design provides a hardware foundation for future closed-loop DBS systems. By integrating real-time neural signal analysis algorithms (e.g., beta-band oscillation detection in Parkinsonian states), this platform could enable adaptive neuromodulation that dynamically adjusts stimulation parameters based on pathological biomarker feedback, thereby enhancing therapeutic precision while reducing side effects.

This research provides a theoretical foundation for targeted precision therapy in neurological disorders such as Parkinson’s disease.

## Figures and Tables

**Table 1:** Tissue physical parameters.

Parameter	Description	Unit	Magnitude	Note
k	Conductivity of tissue	W/(m·°C)	2	
c	Specific heat of tissue	J/(kg·°C)	1800	
ρ	Density of tissue	kg/m^3^	998	
c_b_	Specific heat of blood	J/(kg·°C)	3600	
*w_b_*	Blood perfusion rate	s^−1^	0.0005	
Q_m_	Metabolic heat generation	W/m^3^	4200	
σ	Specific conductivity	S/m	0.33	
**ε**	Permittivity	1	1 × 10^6^	100~1000 Hz

**Table 2:** Simulation condition settings.

X_1_ (I/mA)	X_2_ (f/Hz)	X_3_ (P_w_/μs)
0.1	50	60
0.5	100	100
0.8	150	150
1.0	-	200

## Data Availability

The original contributions presented in this study are included in the article. Further inquiries can be directed to the corresponding author(s).

## Appendix A

The 48 sets of simulation results in Section 3.3 are as follows.

**Table A1:** Results of simulation in Section 3.3.

No.	X_1_ (P_w_/μs)	X_2_ (f/Hz)	X_3_ (I/mA)	Y (VTA/mm^3^)
1	200	150	0.1	10.77
2	200	150	0.5	24.4
3	100	150	1.0	34.51
4	60	100	1.0	24.64
5	150	100	1.0	34.59
6	100	100	0.5	24.37
7	60	100	0.8	21.88
8	200	50	1.0	34.605
9	60	50	0.8	21.89
10	200	100	1.0	34.61
11	60	150	1.0	24.63
12	150	100	0.8	31.01
13	60	50	1.0	24.69
14	150	50	1.0	34.62
15	150	50	0.1	10.71
16	60	100	0.5	17.25
17	100	150	0.5	24.31
18	200	100	0.8	31.03
19	150	150	0.1	10.69
20	150	150	1.0	34.61
21	60	150	0.1	10.65
22	200	50	0.1	10.85
23	100	150	0.8	30.96
24	200	50	0.8	31.04
25	150	50	0.8	31.02
26	100	50	0.8	31.01
27	100	150	0.1	10.68
28	200	150	1.0	34.54
29	100	100	0.8	30.97
30	200	50	0.5	24.41
31	60	50	0.1	10.66
32	60	50	0.5	17.26
33	150	150	0.8	30.99
34	100	100	1.0	34.56
35	100	100	0.1	10.7
36	150	100	0.1	10.78
37	60	150	0.8	21.87
38	200	100	0.1	10.81
39	150	100	0.5	24.38
40	150	150	0.5	24.37
41	100	50	0.1	17.03
42	200	150	0.8	31.02
43	100	50	0.5	24.37
44	60	100	0.1	7.6
45	150	50	0.5	24.39
46	200	100	0.5	24.4
47	60	150	0.5	17.24
48	100	50	1.0	34.58
